# Isolation and Transcriptomic Characterization of Genotype 5 Japanese Encephalitis Virus E138K Mutant Strain

**DOI:** 10.1155/tbed/9733265

**Published:** 2026-05-14

**Authors:** Yuhong Yang, Ruichen Wang, Weijia Zhang, Qikai Yin, Fan Li, Shihong Fu, Kai Nie, Qianqian Cui, Songtao Xu, Huanyu Wang

**Affiliations:** ^1^ National Key Laboratory of Intelligent Tracking and Forecasting for Infectious Diseases, NHC Key Laboratory of Biosafety, Beijing Key Laboratory of Viral Infectious Diseases, National Institute for Viral Disease Control and Prevention, Chinese Center for Disease Control and Prevention, Beijing, 102206, China, chinacdc.cn

**Keywords:** E138K mutation, genotype 5 Japanese encephalitis virus, transcriptomic characterization

## Abstract

The recent emergence of the genotype five Japanese encephalitis virus (G5 JEV) has once again drawn public attention to public health concerns. Plaque assay analysis of G5 JEV parental strain XZ0934 revealed two distinct plaque morphologies. To investigate this phenotypic heterogeneity, we performed single‐plaque purification and isolated two strains, designated as XZ0934‐L (large plaque) and XZ0934‐S (small plaque). Subsequently, deep mutational scanning revealed an amino acid mutation at position 138 (E138K) in the E protein of the XZ0934‐S strain. Viral titer determination and plaque morphology analysis showed that the titers of XZ0934‐L and XZ0934‐S in BHK‐21 cells were 10^7.06^ PFU/mL and 10^7.35^ PFU/mL, respectively, with plaque diameters of 0.87 ± 0.12 mm and 0.38 ± 0.08 mm. However, while both strains induced cytopathic effects across six cell lines used in this study, XZ0934‐S produced markedly weaker CPE than XZ0934‐L in N2a cells. Spatial modeling predicted that the E138K substitution did not significantly alter the overall conformation of the E protein. In contrast, transcriptomic analysis demonstrated that infections with different JEV genotypes induced significantly distinct host gene expression profiles in N2a cells. The XZ0934‐S strain caused the mildest transcriptional perturbations, and the perturbation of regulatory pathways was markedly weaker than those of the XZ0934‐L strain. Previous studies have suggested that the E138K mutation can attenuate the neurovirulence of JEV. This study provides the first comprehensive in vitro characterization of an E138K mutant in G5 JEV. The XZ0934‐S mutant strain, given its small plaque phenotype and reduced transcriptional impact, represents a promising candidate for further vaccine development. Future studies should rigorously evaluate its safety profile, genetic stability, and immunogenicity in animal models.

## 1. Introduction

In 1935, Japanese researchers first isolated a virus from the brain tissue of deceased encephalitis patients and named it Japanese encephalitis virus (JEV) [1]. JEV belongs to the genus *Orthoflavivirus* within the family *Flaviviridae*. Its genome consists of a single‐stranded positive‐sense RNA ~11 kb in length, flanked by 5′ and 3′ untranslated regions (5′‐ and 3′‐UTRs). The genome contains only one open reading frame, which encodes three structural proteins (C, prM, and E) and seven nonstructural proteins (NS1, NS2a, NS2b, NS3, NS4a, NS4b, and NS5) [2]. With the advancements in viral molecular biology theory and technology, it is now possible to classify JEV into five genotypes (G1–G5) based on nucleotide sequences including the prM gene, E gene, whole genome, and even the 3′ UTR [3–6].

The G1–G5 JEV originated from the Indonesia–Malaysia region in Southeast Asia [7, 8]. Before the 1990s, the strains isolated from patients, animal hosts, and mosquitoes in JEV‐endemic regions were predominantly G3. After entering the 2000s, G1 and G3 began to cocirculate. Over the past two decades, G1 has gradually replaced G3 to become the dominant genotype in Asian JE‐endemic regions [9–11]. In contrast, G2 and G4 are mainly distributed in Australia and Papua New Guinea and in eastern Indonesia, respectively [12–14]. G5 has been identified in countries such as Indonesia, China, and South Korea [15].

The JEV genotypes in China exhibit dynamic shifts and diversification trends, which are consistent with global JEV dynamics. In 1949, three strains of JEV isolated from the brain tissue of encephalitis patients were later identified as G3 through molecular biological analysis [16]. Subsequently, the majority of strains isolated in China also belonged to the G3. In 1979, China first isolated G1 JEV from *Culex tritaeniorhynchus* [17]. After 2000, the number of G1 isolates gradually increased, leading to a cocirculation pattern of G1 and G3. In recent years, G1 has replaced G3 in natural circulation to become the dominant JEV genotype in China [9]. Meanwhile, in South Korea, the prevalent JEV genotype in nature shows a shift toward G5 [18]. China has also isolated G5 strains from mosquitoes, suggesting that this genotype may pose a new public health threat.

In 2009, a strain of G5 JEV (XZ0934) was isolated from *Culex tritaeniorhynchus* collected in the Linzhi City of the Xizang Autonomous Region [19]. During the early isolation of this viral strain, two plaque morphologies with different diameters were observed. Following single‐plaque purification, two G5 JEV strains (XZ0934‐L and XZ0934‐S) were successfully obtained, which were then subjected to molecular identification and comparative characterization. Their replication characteristics in six cell lines and transcriptomic responses in infected N2a cells were evaluated. Finally, we identified a mutant G5 strain carrying the E138K mutation. This study therefore not only confirms the presence and phenotypic impact of the E138K mutation in G5 JEV but also provides novel insights into JEV pathogenic mechanisms by uncovering G5‐specific transcriptional signatures.

## 2. Materials and Methods

### 2.1. Viruses and Cells

All virus strains used in this study, NX1889, P3, and XZ0934, were preserved in our laboratory. Among them, NX1889 (G1; GenBank ID: MT134112.1) was isolated in 2018 from a human cerebrospinal fluid sample collected in Yinchuan, Ningxia Hui Autonomous Region [20]. P3 (G3; GenBank ID: AF036919.1) was isolated in 1949 from the brain tissue of a fatal encephalitis case in Beijing. XZ0934 (G5; GenBank ID: JF915894.1) was isolated in 2009 from *Culex tritaeniorhynchus* collected in Linzhi, Xizang Autonomous Region [19].

All cell lines used in this study were obtained from our laboratory’s cell bank. These include rodent cell lines (Baby Hamster Kidney‐21, BHK‐21; Neuro‐2a, N2a), a monkey‐derived cell line (African Green Monkey Kidney Cells, Vero), a mosquito cell line (*Aedes albopictus* clone C6/36, C6/36), a porcine cell line (Porcine Kidney‐15, PK‐15), and an avian cell line (Douglas Foster‐1, DF‐1).

### 2.2. Viral Plaque Purification

The XZ0934 JEV strain was subjected to serial 10‐fold dilutions. One hundred microliters of each dilution was added to BHK‐21 monolayers in 6‐well plates and incubated at 37°C for 1 h. Subsequently, 1% MEM‐agarose semi‐solid overlay medium was added to each well. After solidification, the plate was placed in a 37°C incubator with 5% CO_2_. Cytopathic effects (CPEs) were observed daily under a microscope. When plaques became visible, a second overlay medium containing a neutral red solution was added. Following solidification at room temperature, the plate was returned to the incubator. Distinct plaques were clearly observed the next day.

Individual plaques, clearly isolated from the surrounding ones, were selected. A sterile pipette tip with its end cut off was used to vertically core out and aspirate the agarose plug at the plaque location. The plug was then transferred into 500 µL of the MEM medium. After vortexing, 100 µL of this suspension was used to inoculate the BHK‐21 monolayer in a 6‐well plate. The CPEs were monitored daily. When CPEs reached 70%–80%, both cells and the supernatant from the infected well were harvested. This harvest underwent three cycles of freezing and thawing, followed by centrifugation at 4°C and 2500 × g for 15 min. The clarified viral supernatant was collected. In total, four rounds of plaque purification were performed to obtain viruses with distinct plaque morphologies.

### 2.3. Viral Titer Determination

BHK‐21 cells were seeded into 6‐well plates. When the cells reached ~90% confluence the following day, the viral stock was subjected to serial two‐fold dilutions. Each dilution was inoculated in duplicate onto the cell monolayers (0.1 mL per well). The plates were then incubated at 37°C with 5% CO_2_ for 1 h. After adsorption, 4 mL of maintenance medium consisting of MEM with 1% methylcellulose was added to each well. The plates were returned to the incubator for 4–5 days. When plaques became clearly visible under the microscope, the overlay medium was removed. Each well was stained with 2% crystal violet solution for 30–50 min at room temperature. The plates were gently rinsed and air‐dried. The plaques were then counted to calculate the viral titer [21].

### 2.4. Determination of Viral Growth Curves

Viruses were inoculated into BHK‐21, Vero, C6/36, PK‐15, DF‐1, and N2a cells at an MOI = 0.1. CPEs were observed and recorded every 12 h.

Cells were infected with the XZ0934‐L and XZ0934‐S strains at an MOI = 0.1. After 1 h of adsorption, the cells were washed once with PBS and incubated with the appropriate maintenance medium. Supernatants and cells were collected at 0, 12, 24, 36, and 48 h post‐infection (hpi). Following three freeze–thaw cycles, the samples were centrifuged at 4°C and 2500 × g for 15 min to collect the supernatant. Viral titers in the supernatant were determined by the plaque assay, and growth curves of the virus in different cell lines were plotted using GraphPad Prism software. Each time point was independently measured in triplicate, and the average value was calculated [22].

### 2.5. Full‐Genome Sequencing of G5 JEV

Full‐genome primers for G5 JEV from a previous study were used [19], and new primers were designed for segments that failed to amplify with the published primers. All primers used for full‐genome amplification are listed in Table S1. PCR amplification was performed using the PrimeScript II High Fidelity One‐Step RT‐PCR Kit (Takara, Osaka, Japan). The PCR amplicons were analyzed by 1% agarose gel electrophoresis to verify their expected size. Amplicons of the correct size were purified and sent to DIA‐UP BIOTECH (Tongzhou District, Beijing) for Sanger sequencing. The sequencing results were confirmed by BLAST analysis (https://blast.ncbi.nlm.nih.gov/Blast.cgi) and assembled using the SeqMan software (v7.1.0.44).

### 2.6. Next‐Generation Sequencing and Transcriptome Library Construction

RNA was extracted from the viral samples using a viral DNA/RNA extraction kit (Xi’an Tianlong Technology Co., Ltd., Xi’an, China) on a fully automated nucleic acid extraction system. Next‐generation sequencing (NGS) was performed using the Ovation RNA‐seq System V2 kit (Nugen, San Carlos, CA, USA) and the Nextera series kit (Illumina, San Diego, CA, USA).

Previous studies by our team have shown that BHK‐21, Vero, C6/36, and PK‐15 cells support JEV replication with significant CPE, high viral titers, and high RNA levels. Therefore, these cell lines are well suited for virus isolation and cultivation. In contrast, N2a and DF‐1 cells are more appropriate for investigating JEV pathogenesis [23]. Based on these findings, JEV strains NX1889 (G1), P3 (G3), XZ0934‐L (G5), and XZ0934‐S (G5) with predetermined viral titers were used to infect N2a cells at MOI = 0.1. After 1 h of viral adsorption, cells were washed once with PBS and then maintained in the culture medium. Based on previous growth curve studies [23], in N2a cells, three genotypes of JEV reach peak viral replication at ~48 hpi, with evident CPEs observed at this time point. To capture and compare the stable host transcriptional response differences induced by distinct JEV strains at the peak replication phase, this study collected cells and supernatants at 48 h post infection RNA extraction. For the uninfected control group, N2a cells were treated identically but exposed only to the culture medium without virus. The extracted RNA was subsequently used for transcriptomic library construction and sequencing. Three biological replicates were included for each viral strain and for the uninfected control group.

### 2.7. Structural Modeling of the JEV E Protein

Based on the amino acid sequence, the three‐dimensional structure of the protein was modeled using the AlphaFold 2.0 (v2.3.0) artificial intelligence program. AlphaFold processes the sequence information through feature construction, converting it into a format interpretable by the model [24]. It then employs the Evoformer module to refine multiple sequence alignment (MSA) data and extract pairwise residue features [25]. Finally, the structure module uses this information to predict the three‐dimensional coordinates of each amino acid, thereby constructing the predicted 3D structure of the protein.

### 2.8. Transcriptome Sequencing Analysis

#### 2.8.1. Differential Gene Expression Analysis

Raw sequencing data quality was assessed using the CLC Genomics Workbench (v2.0, QIAGEN, Hilden, Germany). Quality control was performed with Trimmomatic (v0.39) to remove adapter sequences and low‐quality reads [26]. Transcript quantification against the mouse transcriptome reference GRCm39 was conducted using Salmon (v1.4.0) in validateMappings mode with default parameters [27]. The transcripts per million (TPM) values output by Salmon were used as input for differential expression analysis with the R package DESeq2 (v1.42.0) to identify differentially expressed genes (DEGs) between the infected and control group [28]. The following are examples of linguistic variation in the United States:Genes with *p*  < 0.05 and |log2FC| ≥ 1 are considered significantly differentially expressed. The formula for calculating the TPM is as follows:

TPMA=Number of reads mapped to gene ALength of gene A÷Total number of reads mapped to all genes×109

The ggVennDiagram package (v1.5.2) in R was used to generate Venn diagrams and screen for DEGs in N2a cells infected with different genotypes of JEV.

#### 2.8.2. Principal Component Analysis

Principal component Analysis (PCA) reduces the dimensionality of complex data to lower its complexity, extracts principal components, and analyzes the essential characteristics of the data. PCA is a widely used statistical method with broad applications, including data classification and cluster analysis. Similar samples tend to form clusters, and the closer the distance between samples, the higher their similarity.

#### 2.8.3. KEGG Enrichment Analysis of Differentially Expressed Genes

Based on the Kyoto Encyclopedia of Genes and Genomes (KEGG), clusterProfiler R package (v4.10.0) was used to query the functional annotations of DEGs via the org.Mm.eg.db database (v3.12.0) for KEGG enrichment analysis [29]. This analysis identifies metabolic pathways enriched by DEGs, elucidating differences between samples at the metabolic pathway level. The results were visualized using R (v4.3.1).

### 2.9. Genotype‐Wide Sequence Analysis of the E138 Site in JEV E Protein

To assess the conservation and potential functional role of the E138 residue in the JEV E protein across different genotypes, we conducted a comprehensive analysis of publicly available sequence data. All available full‐length E gene sequences of JEV were retrieved from the NCBI GenBank database (https://www.ncbi.nlm.nih.gov/, accessed on February 3, 2026). The raw data were filtered to retain only high‐quality entries with clearly defined genotype, host, isolation year, and geographical origin. After removing duplicate and incomplete sequences, 355 representative sequences were obtained (210 G1, 8 G2, 121 G3, 8 G4, and 8 G5). The E gene sequences of the two G5 strains isolated and identified in this study (XZ0934‐L and XZ0934‐S) were also included, bringing the final dataset to 357 sequences. All sequences were first aligned using MAFFT v7.450, and inter‐genotype nucleotide and amino acid homology of the E protein was further analyzed using MEGA v11.0. Subsequently, mutation analysis of the amino acid at position 138 of the E protein was performed using an online analysis platform (https://sp-app.streamlit.app/Inforamtion) to extract all variation information at this site. For strains with mutations at the E138 position, we further traced their GenBank entries and related published literature to systematically collect and record the reported biological characteristics of these strains, focusing on descriptions related to their virulence phenotypes, such as whether they were explicitly labeled as “attenuated strain,” “vaccine strain,” “small plaque variant,” or were experimentally confirmed to have low neurovirulence in animal models.

## 3. Results

### 3.1. Heterogeneity in Early Isolates of the JEV XZ0934 Strain

**Figure 1:**
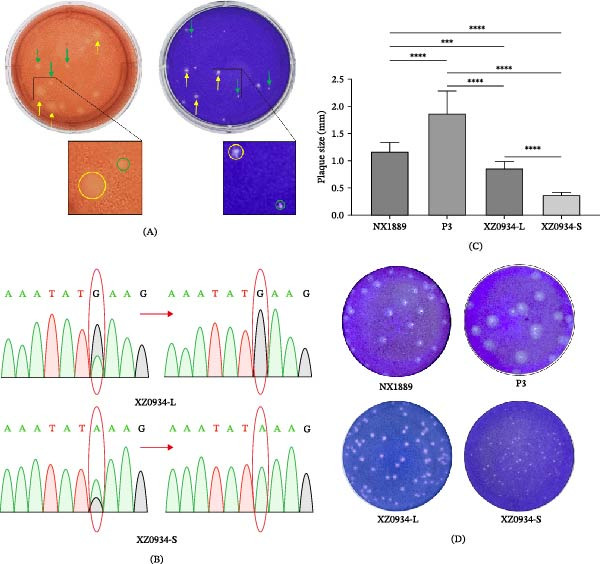
(A) Two distinct plaque morphologies of the original XZ0934 strain. Neutral red staining (left); crystal violet staining (right). The green arrows point to small plaques and the yellow arrows point to large plaques. (B) Sanger sequencing chromatograms confirming the E138K mutation in the purified XZ0934‐S strain. (C,D) Plaque size and morphology of the indicated JEV strains in BHK‐21 cells. Statistical significance was determined by one‐way ANOVA with Tukey’s post hoc test ( ^∗∗∗^ *p*  < 0.001;  ^∗∗∗∗^ *p*  < 0.0001); non‐significant differences are not indicated (*p* ≥ 0.05).

To perform NGS, RNA was extracted from the supernatant of the XZ0934 isolate after three consecutive passages in BHK‐21 cells. The full‐length sequence of the XZ0934 strain (GenBank Accession No. JF915894) was selected as the reference for sequence assembly. The consensus sequence generated from NGS was translated into an amino acid sequence and compared with the amino acid sequence of XZ0934, revealing inconsistencies at three amino acid sites (Table 1). Consistent with this genetic heterogeneity, the initial isolate exhibited heterogeneous plaque morphologies, forming two distinct plaque sizes in BHK‐21 cells (Figure 1A).

**Table 1:** Single nucleotide variants information of the primary isolate of XZ0934 strain.

Region	Reference	Allele	Count	Coverage	Frequency %	Amino acid mutation	Protein region
1148	A	G	450	5991	7.51	—	—
1389	G	A	209	12,121	13.50	E432K	E 138
1893	G	A	635	13,334	4.76	E600K	E 306
2300	G	A	564	6495	8.68	—	—
3545	G	A	3438	14,166	24.30	—	—
7630	C	T	413	5199	7.94	S2512L	NS4b 239

*Note:* “—” Nucleotide mutations at this site did not cause amino acid mutations.

### 3.2. Single Plaque Purification Process of XZ0934‐L and XZ0934‐S Strains

NGS of the strains amplified after two rounds of single‐plaque purification revealed that the virus still exhibited genetic heterogeneity. Therefore, this study conducted two additional rounds of single‐plaque purification. The strain exhibiting a larger plaque morphology was designated as XZ0934‐L, while the strain exhibiting a smaller plaque morphology was designated as XZ0934‐S. The amino acid site differences observed across the four rounds of sequencing are detailed in Table 2. Compared to the reference sequence, the complete genome of the XZ0934‐L strain showed two nucleotide differences that did not result in amino acid changes, while the XZ0934‐S strain exhibited four nucleotide differences and one amino acid change. The key nucleotide differences in both strains were further validated by Sanger sequencing (Figure 1B). Following the identification of this mutation (a nucleotide variation at position 1389 resulting in an E138K substitution in the E protein) in the XZ0934‐S strain, plaque assays of both purified viruses in BHK‐21 cells demonstrated a uniform morphology (Figure 1D), confirming the successful isolation of two distinct G5 JEV strains.

**Table 2:** Single nucleotide variants information of the purified XZ0934‐L and XZ0934‐S strains.

Strain	Region	Reference	Allele	Count	Coverage	Frequency %	Amino acid mutation	Protein region
XZ0934‐L	1148	A	G	11,223	11,287	99.43	—	—
	2300	G	A	8777	8814	99.58	—	—
XZ0934‐S	1389	G	A	16,750	16,877	99.25	E432K	E138
	5546	C	T	16,826	17,218	97.72	—	—
	6398	G	A	11,685	11,743	99.51	—	—
	10,560	C	A	1154	13,050	8.84	—	—

### 3.3. Viral Titer Determination and Plaque Size Analysis

The titers of NX1889, P3, XZ0934‐L, and XZ0934‐S JEV strains amplified in BHK‐21 cells were determined using the plaque assay. The viral titration results are as follows: the titer of the NX1889 strain was 10^6.59^ PFU/mL, the P3 strain was 10^7.01^ PFU/mL, the XZ0934‐L strain was 10^7.06^ PFU/mL, and the XZ0934‐S strain was 10^7.35^ PFU/mL (Figure 1D).

The plaque sizes of the four JEV strains differed. The G1 NX1889 strain exhibited medium‐sized circular plaques with a diameter of 1.18 ± 0.16 mm. The G3 P3 strain displayed the largest circular plaques, measuring 1.88 ± 0.4 mm in diameter. The two G5 viral strains produced the smallest plaques: the XZ0934‐L strain had plaques with a diameter of 0.87 ± 0.12 mm, while the XZ0934‐S strain had plaques measuring 0.38 ± 0.08 mm in diameter (Figure 1C).

### 3.4. Comparison of Virulence and Growth Kinetics of Two G5 JEV Strains in Different Cell Lines

**Figure 2:**
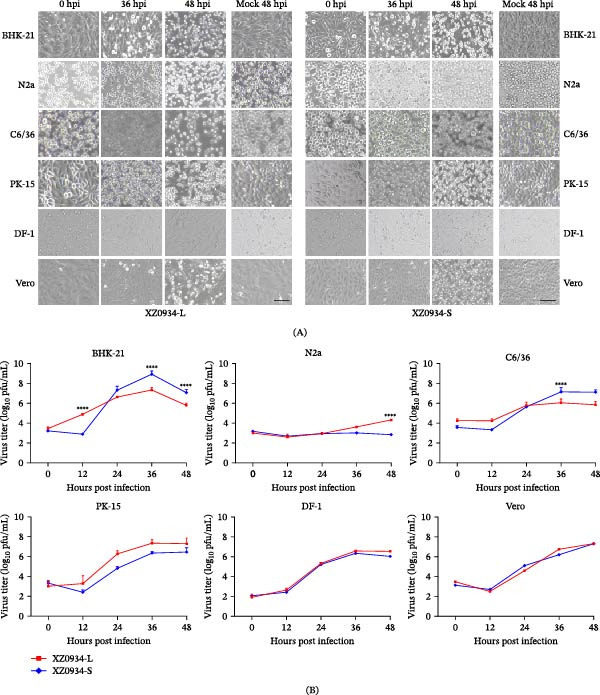
(A) Cytopathic changes observed in different cell lines at 36 and 48 hpi. Cells were infected with XZ0934‐L, XZ0934‐S or mock. Scale bar: 100 µm. (B) Growth curves of two strains (XZ0934‐L and XZ0934‐S) of G5 JEV on different cell lines. Statistical significance between the two strains across all cell lines and time points was determined by two‐way ANOVA followed by Šídák’s multiple comparisons test ( ^∗∗∗∗^ *p*  < 0.0001); non‐significant differences are not indicated (*p* ≥ 0.05).

This study evaluated the replication capacity of two G5 JEV strains (XZ0934‐L and XZ0934‐S) in six cell lines. The results showed that both strains replicated effectively in BHK‐21, Vero, C6/36, DF‐1, N2a, and PK‐15 cells, accompanied by CPE characterized by cell detachment, shrinkage, fragmentation, and death. The CPE intensified with prolonged infection time (Figure 2A). BHK‐21 cells were the most sensitive to viral infection, with CPE appearing at 36 hpi. Notably, in N2a cells, the CPE induced by XZ0934‐S was markedly milder than that induced by XZ0934‐L.

To further assess viral replication capacity, the growth kinetics of both strains in the six cell lines was analyzed (Figure 2B). The results revealed distinct replication patterns between XZ0934‐L and XZ0934‐S. In BHK‐21 cells, XZ0934‐S exhibited faster replication kinetics and higher peak viral titers, whereas in N2a cells, the replication capacity of XZ0934‐L was significantly superior to that of XZ0934‐S. In C6/36 cells, XZ0934‐S displayed elevated replication levels during the late infection stage. However, in PK‐15, DF‐1, and Vero cells, both strains exhibited generally similar replication trends, though XZ0934‐L maintained a slight advantage in replication kinetics and the final titer.

### 3.5. Structural Analysis of the E138 Site in G5 JEV E Protein and Comparative Analysis of E Gene Sequences Across Genotypes

The E gene sequences of the two virus strains obtained through Sanger sequencing were compared with the E genes of all G5 JEV strains deposited in GenBank. The comparison revealed that only XZ0934‐S exhibited a nucleotide change from G to A at position 1389, resulting in an amino acid substitution from glutamic acid (E) to lysine (K) at position 138 of the E protein (Figure S1A). Further analysis of the E‐protein structure of XZ0934‐S revealed that this single amino acid substitution did not significantly alter its overall conformation (Figure S1B). The amino acid positions corresponding to each domain are detailed in Figure S1C.

To further assess the conservation and functional significance of the E138 residue across all JEV genotypes, we analyzed 357 complete E gene sequences. The results confirmed that E138 was highly conserved across all five JEV genotypes, with mutations observed in only 23 sequences (6.40%), including 2 substitutions to neutral amino acids (A/G) and 21 substitutions to basic amino acids (K/R) (Table S3). Among these, six of the substitutions to basic amino acids have been confirmed as attenuated variants [30–35]. Notably, comparative analysis revealed that the nucleotide sequence homology between G5 and G1–G4 (77.37%–78.16%) was substantially lower than that among G1–G4 themselves (82.57%–89.70%), indicating a distinct evolutionary and structural context for G5 (Table S2).

Prior to this study, all reported attenuated mutants with E138K/R substitutions belonged exclusively to G1 or G3, and no G5 strain carrying such a mutation had ever been documented. Therefore, the identification of the E138K mutation in G5 isolate XZ0934‐S, coupled with its associated small plaque phenotype, provides the first direct evidence that this conserved attenuation mechanism may remain functional within the genetically distinct G5 background. This finding not only extends the known biological relevance of E138K from G1/G3 to G5 but also offers novel insights into genotype‐specific virulence regulation in JEV.

### 3.6. Transcriptomic Characterization of N2A Cells Infected With Different JEV Genotypes

#### 3.6.1. Analysis of Gene Expression Profiles

Transcriptomic sequencing data were used to analyze the gene expression profiles of N2a cells infected with different JEV genotypes. The detailed transcript abundance across all samples, expressed in TPM values, is provided in Table S4. The TPM boxplot (Figure S2A) demonstrated high consistency in gene expression levels both between and within groups—comprising the four virus‐infected groups (NX1889, P3, XZ0934‐S, and XZ0934‐L) and the control group—indicating strong experimental reproducibility and data reliability. Gene expression density distribution analysis (Figure S2B) further confirmed that all samples exhibited typical normal distribution characteristics. Compared with the control group, gene expression abundance was generally reduced in each infected group, with highly consistent expression trends observed within groups. These findings indicate that viral infection induced systematic transcriptomic alterations and further confirm the stability of experimental conditions and the good comparability of the samples.

#### 3.6.2. Comparative Analysis of Transcriptomic Features Induced by Different JEV Genotypes

**Figure 3:**
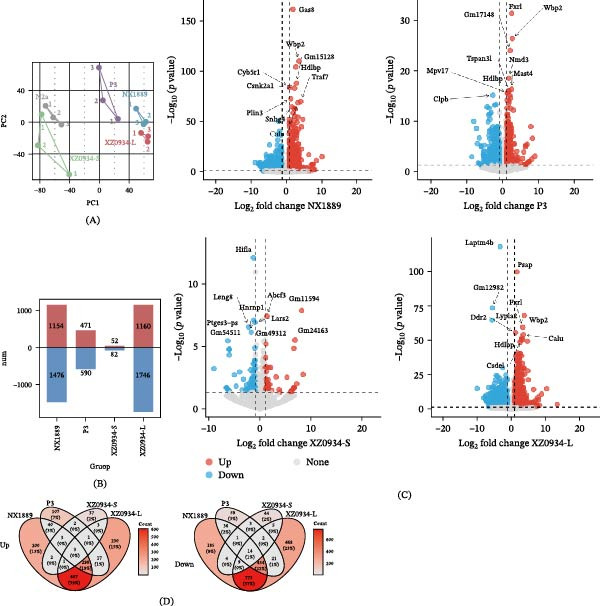
Transcriptomic analysis of N2a cells infected with different JEV genotypes for 48 h. (A) PCA (Principal component analysis). The abscissa denotes the first principal component (PC1), and the ordinate denotes the second principal component (PC2). Different groups (NX1889, P3, XZ0934‐S, XZ0934‐L) and their three replicates are labeled. (B) Bar plot of the number of DEGs across groups (NX1889, P3, XZ0934‐S, XZ0934‐L). Red bars represent up‐regulated genes, blue bars represent down‐regulated genes, and the numbers above bars indicate the count of DEGs in each category. (C) Volcano plots of DEGs in NX1889, P3, XZ0934‐S, and XZ0934‐L groups. The abscissa is log2(Fold Change), and the ordinate is ‐log10(*p*‐value). Red dots indicate up‐regulated DEGs, blue dots indicate down‐regulated DEGs, and gray dots represent non‐significant genes. Representative DEGs in each group are labeled. (D) Venn diagrams of specific up‐regulated (upper) and down‐regulated (lower) genes among NX1889, P3, XZ0934‐S, and XZ0934‐L groups. Numbers in each segment represent gene counts, with percentages (relative to the total in the segment) in parentheses; the color scale on the right indicates gene count density.

The PCA of transcriptomic features induced by different JEV genotypes revealed distinct clustering patterns (Figure 3A). Detailed PCA data are provided in Table S6. The infected groups showed clear separation in the PCA, with the transcriptional profile of the XZ0934‐S‐infected group being closer to that of the control group. In contrast, the NX1889, P3, and XZ0934‐L‐infected groups exhibited greater transcriptional divergence, visually demonstrating significant differences in host cell transcriptional responses elicited by the different viral genotypes.

Statistical results of DEGs indicated that the XZ0934‐L‐infected group exhibited the most pronounced transcriptomic perturbation, with a total of 2,906 DEGs identified (1,160 up‐regulated and 1,746 down‐regulated) (Figure 3B). The NX1889‐infected group ranked second, with 2,630 DEGs (1,154 up‐regulated and 1,476 down‐regulated), followed by the P3‐infected group with 1,061 DEGs (471 up‐regulated and 590 down‐regulated). In contrast, the XZ0934‐S‐infected group showed only 134 DEGs (52 up‐regulated and 82 down‐regulated). Complete DEGs data are provided in Table S5. The corresponding volcano plots further illustrate the gene expression changes in each group (Figure 3C). The results showed that the NX1889‐ and XZ0934‐L‐infected groups exhibited higher significance in differential expression and a broader enrichment of highly significant genes, with a wider distribution of gene expression fold changes. The P3‐infected group displayed an intermediate level in both the number and significance of DEGs, while the XZ0934‐S‐infected group showed the lowest overall significance in differential expression, the fewest highly significant genes, and relatively concentrated expression changes, with no genes exhibiting extreme expression deviations.

Venn diagrams revealed the specific transcriptional characteristics associated with different viral strains (Figure 3D). Among up‐regulated genes, the NX1889 group contained 200 unique genes, the P3 group had 107 unique genes, and 3 genes were uniquely shared between the XZ0934‐L and XZ0934‐S groups. Only 3 genes were commonly up‐regulated across all four groups. Among down‐regulated genes, the NX1889 group contained 185 unique genes, the P3 group had 59 unique genes, and 5 genes were uniquely shared between the XZ0934‐L and XZ0934‐S groups. Fourteen genes were commonly down‐regulated across all four groups. These findings clearly demonstrate significant differences in transcriptional responses induced by infection with different JEV strains.

#### 3.6.3. KEGG Pathway Enrichment Analysis

Based on the screened DEGs, KEGG pathway enrichment analysis was further conducted to assess their potential functions. The key pathways enriched by the most significantly up‐regulated and down‐regulated genes were visualized using bubble plots. The results indicated that NX1889 and XZ0934‐L infections induced significant changes in multiple pathways, while P3 affected only a small number of pathways. XZ0934‐S infection did not induce significant enrichment of any KEGG pathways for either up‐regulated or down‐regulated genes. The complete results of the KEGG enrichment analysis are detailed in Table S7.

**Figure 4:**
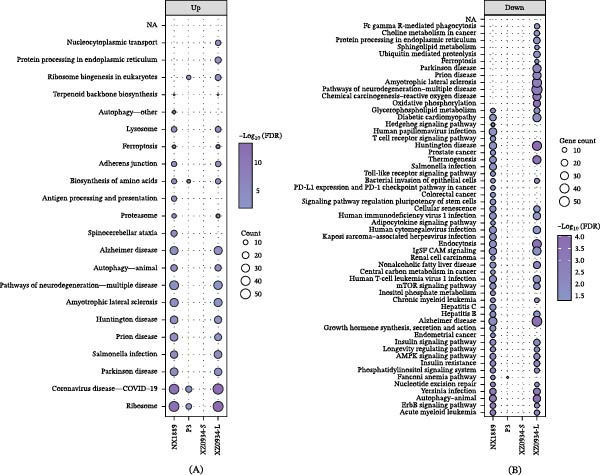
KEGG pathway enrichment analysis of up‐regulated and down‐regulated differentially expressed genes in NX1889, P3, XZ0934‐S, and XZ0934‐L. (A) Up‐regulated pathways; (B) Down‐regulated pathways. Bubbles represent the number of enriched genes in each pathway, and colors indicate the ‐log10(FDR) (false discovery rate‐adjusted *p* value).

Analysis of up‐regulated pathways revealed significant heterogeneity in enrichment characteristics among different viral strains (Figure 4A). NX1889 and XZ0934‐L infection induced perturbations in 19 pathways, respectively. Notably, several pathways related to cellular stress were significantly enriched, including Ribosome, COVID‐19 disease—COVID‐19, Parkinson disease, Pathways of neurodegeneration—multiple diseases, Autophagy—animal, Ferroptosis, and Lysosome. P3 infection was specifically enriched in Ribosome biogenesis in eukaryotes, biosynthesis of amino acids, COVID‐19 disease—COVID‐19, and Ribosome. In contrast, XZ0934‐S infection did not enrich any significantly up‐regulated KEGG pathways. These differences suggest that different viral strains may mediate varying degrees of cellular stress responses and pathological damage by activating distinct up‐regulated pathway networks.

Analysis of down‐regulated pathways showed that XZ0934‐L infection resulted in a broad coverage of down‐regulated pathways, with significant changes in 38 pathways (Figure 4B). These included immune regulatory pathways (such as Fc gamma R‐mediated phagocytosis) and neurodegenerative disease pathways (such as Alzheimer disease and Parkinson disease), among others. The down‐regulated pathways in NX1889 infection were concentrated in immune response and neural function, including the T cell receptor (TCR) signaling pathway and Toll‐like receptor (TLR) signaling pathway. P3 infection exhibited very few corresponding bubbles, with only the Fanconi anemia pathway showing weak enrichment. Similarly, XZ0934‐S infection did not enrich any significantly down‐regulated KEGG pathways. These differences suggest that different viral strains may influence physiological processes such as immune responses, cellular metabolism, and neurological functions by inhibiting distinct down‐regulated pathway networks, thereby contributing to varying infection pathologies.

**Figure 5:**
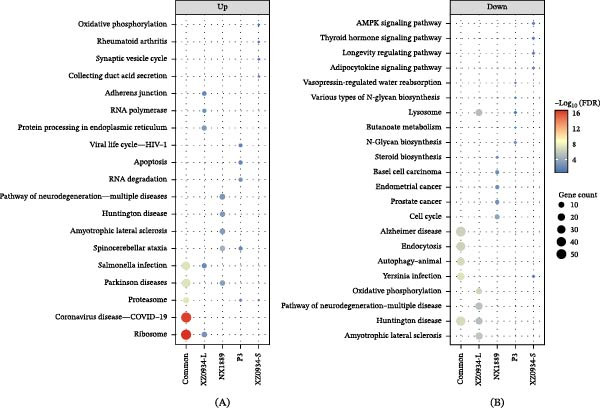
KEGG pathway enrichment analysis of common and strain‐specific differentially expressed genes in JEV‐infected N2a cells. (A) Up‐regulated pathways; (B) Down‐regulated pathways. Bubbles represent the number of enriched genes in each pathway, and colors indicate the ‐log10(*p*‐value).

To elucidate the conserved host response to JEV infection, we performed KEGG pathway analysis on the commonly dysregulated genes across all strains (Table S8). The common up‐regulated DEGs were dramatically enriched in “Ribosome,” suggesting that JEV globally hijacks the host translational machinery to facilitate viral protein synthesis (Figure 5A). Conversely, the common down‐regulated genes were significantly associated with “Autophagy” and various signaling pathways, indicating a universal viral strategy to suppress host innate immune clearance and autophagic degradation (Figure 5B).

Beyond the shared transcriptional responses, we further investigated strain‐specific pathways to understand their distinct pathotypes. Strikingly, the unique up‐regulated genes in the NX1889‐infected group were dominantly enriched in neurodegenerative pathways, including “Spinocerebellar ataxia” and “Parkinson disease,” implying a specific neurotoxic mechanism driven by this strain (Figure 5A). In contrast, XZ0934‐L uniquely induced severe mitochondrial dysfunction and ER stress, evidenced by the significant downregulation of “Oxidative phosphorylation” and upregulation of “Protein processing in endoplasmic reticulum” (Figure 5B). Interestingly, the P3 strain specifically triggered “Apoptosis” pathways (Figure 5A). Together, these unique pathway enrichments potentially explain the differential virulence and neuropathological features among the JEV strains.

## 4. Discussion

In this study, plaque morphology heterogeneity was observed during the amplification of a G5 JEV strain (XZ0934) isolated from mosquito specimens collected in Linzhi City of the Xizang Autonomous Region. Through sequencing and successive single‐plaque purification, two viral strains exhibiting distinct plaque phenotypes were successfully isolated and designated as XZ0934‐L (large plaque) and XZ0934‐S (small plaque), respectively. Sequence analysis revealed that the XZ0934‐S strain carries the E138K mutation in the E protein.

Residue E138 is located in the hinge region at the interface between domains I and II of the E protein (Figure S1B). Mutations at this site are known to contribute to the attenuated phenotype of the JEV live‐attenuated vaccine strain SA14‐14−2, and studies on G1 and G3 JEV strains have confirmed that mutations at this site can attenuate neurovirulence [36–38]. A single amino acid substitution from glutamic acid (E) to lysine (K) has been shown to reduce the neurovirulence of JEV, and mutations to other basic amino acids similarly result in reduced virulence [30, 39]. In this study, the XZ0934‐S strain was found to carry an identical E→K mutation at residue E138. To further investigate the molecular mechanism by which the E138K mutation affects viral virulence, we constructed a three‐dimensional structural model of the E protein. The results showed that the E138K mutation did not significantly alter the overall spatial conformation of the E protein.

Previous studies have shown that in N2a cells, G5 strains replicate to significantly lower levels than G1 and G3 strains, indicating weaker neurocellular adaptability [23]. This observation is consistent with earlier speculations based on differences in E‐protein sequences [40, 41]. In this study, N2a cells were further utilized to investigate the mechanisms underlying JEV‐induced neurocyte damage. The selection of N2a cells as a model for studying JEV neuropathogenesis is well supported by prior research. Ghosh et al. demonstrated that JEV infection in N2a cells induces upregulation of host proteins such as DJ‐1, which modulates viral replication, interferon responses, and lipid receptor expression—key processes in neurotropic viral infection. Furthermore, N2a cells are amenable to transcriptomic profiling and genetic manipulation, making them a suitable system for dissecting genotype‐specific host responses [42]. Our use of this cell line thus builds upon established models to explore differential transcriptional reprogramming induced by distinct JEV strains. In this study, transcriptomic analysis of N2a cells infected with four JEV strains revealed significant heterogeneity in the host transcriptional responses elicited by viruses of different genotypes. The two G5 strains (XZ0934‐L and XZ0934‐S) exhibited markedly distinct transcriptional perturbation profiles: the XZ0934‐L strain triggered the most extensive gene expression changes, whereas the mutant strain induced the mildest transcriptional alterations. Compared with XZ0934‐L (G5), the G1 strain NX1889 also elicited broad transcriptional reprogramming, whereas the G3 strain P3 induced an intermediate level of perturbation, falling between those of NX1889 and XZ0934‐S.

At the signaling pathway level, the differential regulation observed across strains provides a mechanistic link to their divergent pathological phenotypes. The strong activation of cellular stress‐ and neuropathology‐related pathways by the G5 (XZ0934‐L) and G1 (NX1889) strains correlates with their ability to induce significant CPE in N2a cells. For instance, the up‐regulation of the ferroptosis pathway is consistent with the studies showing that JEV triggers iron‐dependent lipid peroxidation and mitochondrial damage in neurons, directly contributing to cell death [43]. The upregulation of neurodegeneration‐related pathways (e.g., Parkinson disease and Alzheimer disease) aligns with the recent finding that the JEV NS4A protein disrupts mitochondrial quality control through its interaction with PINK1 [44]. The concurrent upregulation of the autophagy pathway further supports this link between JEV infection and neuronal damage: while JEV‐induced autophagy suppresses innate immunity and promotes viral replication, its dysregulation results in impaired mitochondrial clearance, increased oxidative damage, and elevated cell death [45]. Finally, upregulation of ribosome‐related pathways reflects virus‐induced translational stress, where the host’s protein synthesis machinery is hijacked to produce viral proteins, starving the cell of factors needed for survival and repair [46].

Notably, NX1889 specifically down‐regulated immune‐ and neural function‐related pathways, including the TCR and TLR signaling pathway. The down‐regulation of the TCR pathway, which is critical for T cell activation and adaptive immunity, has been linked to impaired immune surveillance and increased susceptibility to viral infections [47]. Similarly, the suppression of TLR signaling, a key component of innate immunity involved in pathogen recognition and inflammatory responses, may facilitate viral evasion and contribute to neuroinflammation or neuronal dysfunction [48]. In contrast, the down‐regulation of the AMPK/mTOR signaling pathway was observed in both NX1889 and XZ0934‐L infections. Given the central role of this pathway in regulating cellular energy homeostasis, metabolism, and autophagy, its suppression may reflect a common viral strategy to modulate host metabolic processes and support viral replication [49]. It should be noted that the transcriptomic analysis in this study was conducted only at 48 hpi. This time point was chosen based on prior growth kinetics studies and represents the peak replication phase, allowing effective comparison of host transcriptional responses across strains. However, as this single time point cannot capture the temporal dynamics of gene expression, time course studies are necessary in the future.

This study reveals, from a transcriptomic perspective, the complexity and diversity of host responses induced by the JEV infection. Such variation exists not only across genotypes but is also markedly evident between strains of differing virulence within the same genotype. These findings provide new insights into the pathogenic mechanisms of JEV. Although global surveillance has reported relatively few G5 JEV cases, the XZ0934‐L strain identified in this study exhibited the strongest transcriptional perturbation, indicating a significant potential to cause cellular damage. This indicates that the threat posed by G5 JEV should not be underestimated based solely on its currently limited epidemiological spread. Furthermore, NX1889, known for its high epidemic potential [20], also demonstrated broad pathway perturbations, reinforcing the notion that intense host transcriptional reprogramming may be associated with high viral transmissibility and pathogenicity. Conversely, the XZ0934‐S strain, carrying the key E138K mutation, induced uniquely mild transcriptional alterations. Given the limited efficacy of existing vaccines against G5 JEV [50], the in vitro phenotype of XZ0934‐S positions it as a candidate for further vaccine development. However, this proposition remains preliminary and requires full in vivo validation through comprehensive animal studies that assess its genetic stability, attenuation (neurovirulence/neuroinvasiveness), immunogenicity, and protective efficacy against the G5 JEV challenge. Notably, the E138K mutation in XZ0934‐S arose naturally during plaque purification, providing a near‐isogenic comparison with XZ0934‐L. While this offers correlative evidence for its role in attenuation, direct causality will require future reverse genetics studies to confirm. Future work must therefore prioritize these essential preclinical evaluations to determine the translational potential of XZ0934‐S.

## 5. Conclusion

Through the single‐plaque purification of the parental G5 JEV strain XZ0934, we obtained a small‐plaque variant, designated XZ0934‐S, carrying a lysine substitution at position 138 (E138K) in the E protein. This variant exhibited weaker growth kinetics in N2a cells compared to the large‐plaque variant (XZ0934‐L). Transcriptomic analysis further revealed that XZ0934‐S induced less pronounced transcriptional perturbations in host cells than XZ0934‐L and the virulent strain NX1889. Moreover, its transcriptional profile more closely resembled that of vaccine strain P3. Collectively, XZ0934‐S exhibits a small‐plaque phenotype in vitro, induces low‐level cellular perturbation, and shares a key attenuation site with the known vaccine strain SA14‐14−2. These findings provide preliminary evidence warranting further investigation of this strain. However, further evaluation of XZ0934‐S as a potential vaccine candidate will require comprehensive studies in appropriate animal models to assess its genetic stability, safety profile, immunogenicity, and protective efficacy.

## Author Contributions

Conceptualization: Yuhong Yang, Ruichen Wang, Weijia Zhang, and Huanyu Wang. Methodology: Yuhong Yang, Ruichen Wang, and Weijia Zhang. Supervision: Huanyu Wang. Project administration: Fan Li, Qikai Yin, and Huanyu Wang. Visualization: Fan Li, Shihong Fu, Kai Nie, Qianqian Cui, and Songtao Xu. Writing ‐ original draft preparation: Yuhong Yang, Ruichen Wang, and Weijia Zhang. Writing review and editing: Huanyu Wang.

## Funding

This work was supported by the National Key Research and Development Program of China (2023YFC2306001) and the Beijing Natural Science Foundation (L252074).

## Disclosure

All authors reviewed and approved the submitted article.

## Conflicts of Interest

The authors declare no conflict of interest.

## Supporting Information

Additional supporting information can be found online in the Supporting Information section.

## Supporting information


**Supporting Information** Figure S1. (A) Nucleotide and amino acid differences at the E138 site in G5 JEV. (B) Predicted molecular model of the E protein for XZ0934‐S strain, with domain I (yellow), domain II (red), domain III (blue), and the transmembrane region (green). a‐Spatial structure of glutamate (Glu) residues at E138 site of XZ0934‐L strain; b‐Spatial structure of lysine (Lys) residues at E138 site of XZ0934‐S strain. (C) Amino acid site information of the domain of E protein. Figure S2. (A) Distribution map of sample TPM BOX. (B) Density map of gene expression value. Table S1. The supplemented primer information for genome sequencing of G5 JEV. Table S2. Nucleotide and amino acid homology of the JEV E protein among different genotypes. Table S3. Analysis of the E138 mutation. Table S4. Transcript abundance of genes across different samples. Table S5. Differential gene expression analysis. Table S6. Principal component analysis. Table S7. KEGG pathway enrichment analysis of up‐regulated and down‐regulated differentially expressed genes in NX1889, P3, XZ0934‐S, and XZ0934‐L. Table S8. KEGG pathway enrichment analysis of common and strain‐specific differentially expressed genes in JEV‐infected N2a cells.

## Data Availability

The data that support the findings of this study are available from the corresponding author upon reasonable demand.
