# Supplementary material for: Isolation and Transcriptomic Characterization of Genotype 5 Japanese Encephalitis Virus E138K Mutant Strain
Source: Transbound Emerg Dis. 2026 May 14;2026:9733265. doi: 10.1155/tbed/9733265 (PMC13173371; doi:10.1155/tbed/9733265)
Supplement: Supplementary file 1 — Supporting Information Figure S1. (A) Nucleotide and amino acid differences at the E138 site in G5 JEV. (B) Predicted molecular model of the E protein for XZ0934‐S strain, with domain I (yellow), domain II (red), domain III (blue), and the transmembrane region (green). a‐Spatial structure of glutamate (Glu) residues at E138 site of XZ0934‐L strain; b‐Spatial structure of lysine (Lys) residues at E138 site of XZ0934‐S strain. (C) Amino acid site information of the domain of E protein. Figure S2. (A) Distribution map of sample TPM BOX. (B) Density map of gene expression value. Table S1. The supplemented primer information for genome sequencing of G5 JEV. Table S2. Nucleotide and amino acid homology of the JEV E protein among different genotypes. Table S3. Analysis of the E138 mutation. Table S4. Transcript abundance of genes across different samples. Table S5. Differential gene expression analysis. Table S6. Principal component analysis. Table S7. KEGG pathway enrichment analysis of up‐regulated and down‐regulated differentially expressed genes in NX1889, P3, XZ0934‐S, and XZ0934‐L. Table S8. KEGG pathway enrichment analysis of common and strain‐specific differentially expressed genes in JEV‐infected N2a cells. [file TBED-2026-9733265-s001.zip › Supplementary Material/Supporting Information.docx]

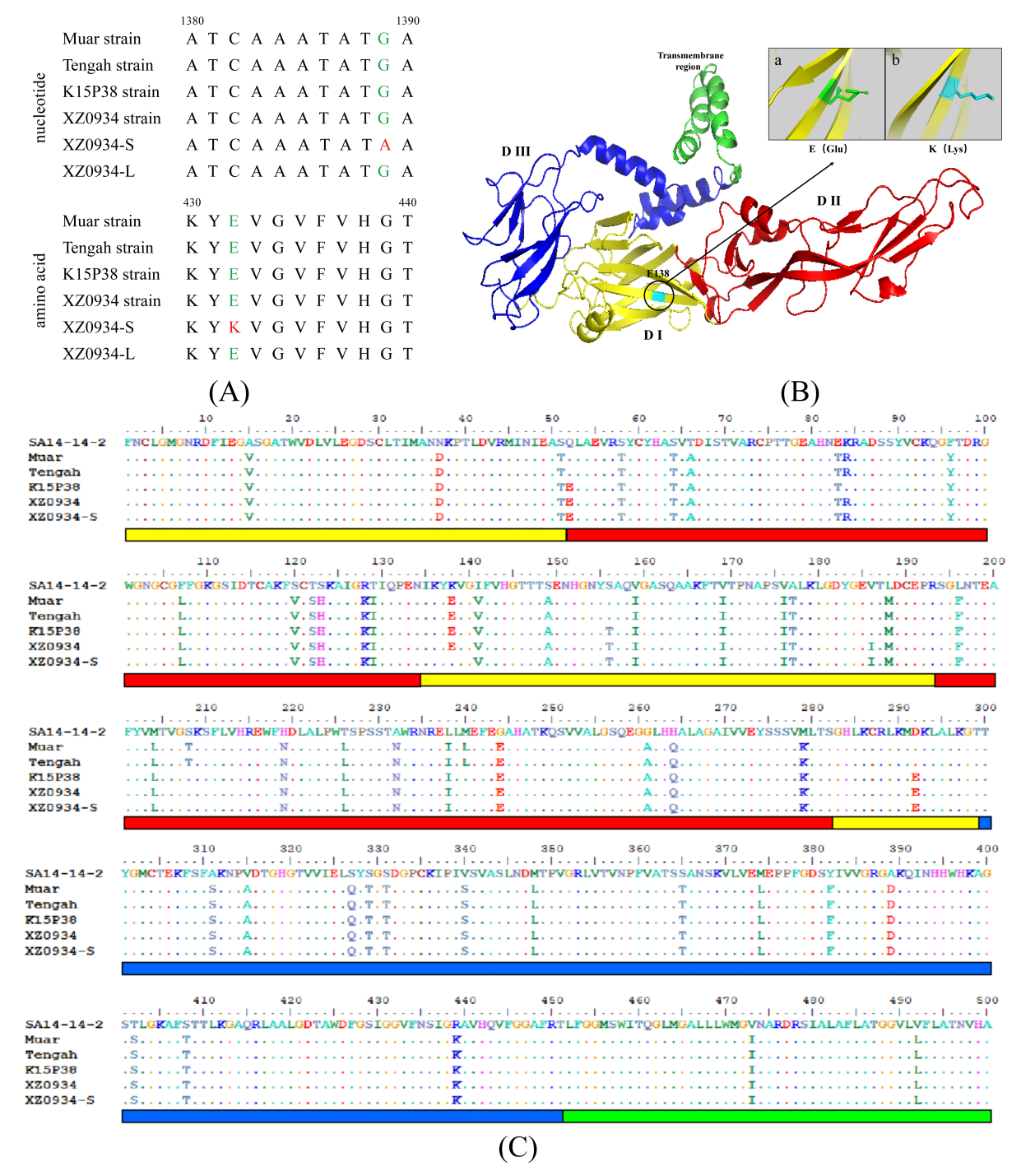


Figure S1. (A) Nucleotide and amino acid differences at the E138 site in G5 JEV. (B) Predicted molecular model of the E protein for XZ0934-S strain, with domain I (yellow), domain II (red), domain III (blue), and the transmembrane region (green). a-Spatial structure of glutamate (Glu) residues at E138 site of XZ0934-L strain; b-Spatial structure of lysine (Lys) residues at E138 site of XZ0934-S strain. (C) Amino acid site information of the domain of E protein.


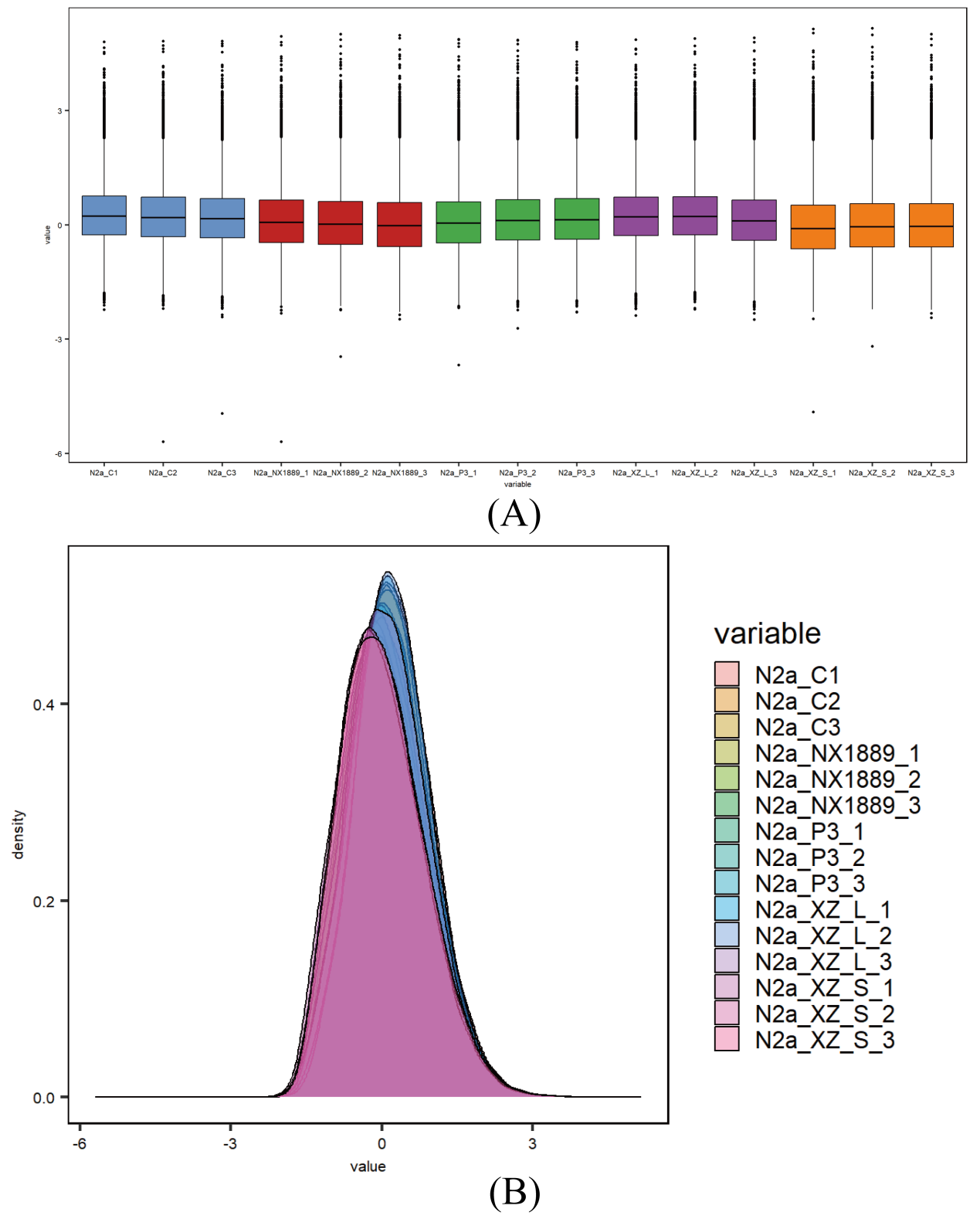


Figure S2. (A) Distribution map of sample TPM BOX. (B) Density map of gene expression value.

Table S1. The supplemented primer information for genome sequencing of G5 JEV.

| Primers | Sequence（5’– 3’） | Position |
| --- | --- | --- |
| S1-F | AGAAGTTTATCTGTGTGAACTTCT | 1-320 |
| S1-R | GCTCTTCTCTACTGCCTTCC |  |
| S2-F | TATGAATGCCCAAAGCTTGA | 627-1686 |
| S2-R | CTCTATTTCTCCAATTTGTGCT |  |
| S3-F | CTAAGTCGTTCTTAGTCCATC | 1600-2627 |
| S3-R | GTGTGCCTTGTGAACTATTT |  |
| S4-F | GGAGATGGCTCATATGTGA | 5076-5922 |
| S4-R | CTCCTTCTTCCAGAATGGT |  |
| S5-F | TTTGTCATCACTACGGACAT | 5820-6776 |
| S5-R | TATTTTCGTCCCTGAGACCT |  |
| S6-F | GCTCATAAGTAAACCCTGG | 8663-9530 |
| S6-R | GTTTGTGAAGGTGTTGAGAG |  |
| S7-F | TTGTCAAAGTAATGAGGCCA | 9415-10299 |
| S7-R | GGTTTATTGCTGCATAGATG |  |
| S8-F | TGAATGGATGAGAGACAAGAC | 10157-10983 |
| S8-R | AGATCCTGTGTTCTTCCTC |  |

Table S2. Nucleotide and amino acid homology of the JEV E protein among different genotypes.

| JEV  Genotypes | JEV Genotypes | | | | |
| --- | --- | --- | --- | --- | --- |
|  | G1 | G2 | G3 | G4 | G5 |
| G1 | *** | 99.03% | 98.32% | 95.35% | 91.47% |
| G2 | 88.04% | *** | 98.46% | 95.30% | 91.32% |
| G3 | 89.70% | 88.71% | *** | 95.11% | 91.49% |
| G4 | 82.57% | 83.16% | 83.10% | *** | 91.36% |
| G5 | 77.37% | 78.16% | 77.96% | 78.03% | *** |

*Note:* “***” indicates 100%; the upper right quadrant indicates the nucleotide similarity; the lower left quadrant indicates the amino acid similarity.
